# Beyond *Streptococcus mutans*: Dental Caries Onset Linked to Multiple Species by 16S rRNA Community Analysis

**DOI:** 10.1371/journal.pone.0047722

**Published:** 2012-10-16

**Authors:** Erin L. Gross, Clifford J. Beall, Stacey R. Kutsch, Noah D. Firestone, Eugene J. Leys, Ann L. Griffen

**Affiliations:** 1 Division of Oral Biology, College of Dentistry, The Ohio State University, Columbus, Ohio, United States of America; 2 Division of Pediatric Dentistry and Community Oral Health, College of Dentistry, The Ohio State University, Columbus, Ohio, United States of America; 3 Department of Dentistry, Nationwide Children’s Hospital, Columbus, Ohio, United States of America; Virginia Commonwealth University, United States of America

## Abstract

Dental caries in very young children may be severe, result in serious infection, and require general anesthesia for treatment. Dental caries results from a shift within the biofilm community specific to the tooth surface, and acidogenic species are responsible for caries. *Streptococcus mutans*, the most common acid producer in caries, is not always present and occurs as part of a complex microbial community. Understanding the degree to which multiple acidogenic species provide functional redundancy and resilience to caries-associated communities will be important for developing biologic interventions. In addition, microbial community interactions in health and caries pathogenesis are not well understood. The purpose of this study was to investigate bacterial community profiles associated with the onset of caries in the primary dentition. In a combination cross-sectional and longitudinal design, bacterial community profiles at progressive stages of caries and over time were examined and compared to those of health. 16S rRNA gene sequencing was used for bacterial community analysis. *Streptococcus mutans* was the dominant species in many, but not all, subjects with caries. Elevated levels of *S. salivarius, S. sobrinus*, and *S. parasanguinis* were also associated with caries, especially in subjects with no or low levels of *S. mutans*, suggesting these species are alternative pathogens, and that multiple species may need to be targeted for interventions. *Veillonella*, which metabolizes lactate, was associated with caries and was highly correlated with total acid producing species. Among children without previous history of caries, *Veillonella*, but not *S. mutans* or other acid-producing species, predicted future caries. Bacterial community diversity was reduced in caries as compared to health, as many species appeared to occur at lower levels or be lost as caries advanced, including the *Streptococcus mitis* group, *Neisseria*, and *Streptococcus sanguinis*. This may have implications for bacterial community resilience and the restoration of oral health.

## Introduction

Dental caries is the most common chronic disease of childhood [1]. It can occur in very young children, shortly after the eruption of teeth, and may be severe. For many children, early childhood caries is a source of pain and impaired quality of life, and for some it results in serious infection, hospitalization, and even fatality [2]. In this young age cohort treatment must often be completed under general anesthesia, accounting for a disproportionate fraction of total dental expenditures [3].

It is of particular importance to understand the microbial etiology of the onset of caries, since preventive interventions such as probiotics or vaccines will be most effective if they interrupt the process before irreversible damage is done to teeth. Once lesions advance beyond the white spot stage and the enamel surface is damaged, they cannot be biologically reversed. Moreover, the disease process may be refractory to ordinary preventive measures that involve biofilm removal such as tooth brushing, since the biofilm becomes more protected from mechanical disruption. Also, early caries experience appears to predispose to greater caries experience later in life, affecting the permanent dentition [4]–[7].

Recent advances, including data from the Human Microbiome Project, have lead to a new paradigm for understanding chronic, bacterially mediated diseases. Diseases of the oral cavity occur in a complex host-bacterial community interaction that often does not fit a single microbe pathogenesis model. Dental caries occurs as the result of a shift in the composition of a biofilm community specific to the human tooth surface. Frequent carbohydrate intake can disrupt the ecology of this community by the selection of acidogenic and acid tolerant species, and these acidogenic communities are responsible for caries development [8], [9]. *Streptococcus mutans* appears to be the most common acid producer in caries initiation [10], but *S. mutans* is not present in all children with caries, and when found it is part of a complex microbial community [11]–[15]. Understanding the degree to which multiple acidogenic species provide functional redundancy and resilience to caries-associated communities is important for developing biologic interventions. Additionally the importance of microbial community interactions in caries pathogenesis is not well understood, including the contribution of bacterial community members in promoting health, such as alkali production [16] or colonization resistance.

Technical advances in 16S rRNA gene analysis have made it possible to comprehensively examine the composition of microbial communities and to study differences between health and disease. The purpose of this study was to investigate bacterial community profiles associated with the onset of early childhood caries in the young primary dentition, and to compare them to bacterial communities found on healthy teeth and in dentally healthy children. In a combination cross-sectional and longitudinal design, bacterial community profiles at progressive stages of caries and over time were examined. 16S rRNA gene cloning and sequencing were used for bacterial community analysis. *Streptococcus mutans* was found to be the dominant species in many, but not all, subjects with caries; elevated levels of *Streptococcus vestibularis/Streptococcus salivarius, Streptococcus sobrinus*, and *Streptococcus parasanguinis* were also associated with caries, especially in subjects with no or low levels of *S. mutans*, suggesting these species are alternative pathogens. Bacterial community diversity was reduced in caries as compared to health, as many species appeared to occur in reduced numbers or be lost as caries advanced. This may have implications for bacterial community resilience and restoration of oral health.

## Results

### Demographics and Clinical Outcomes

Thirty-six subjects with caries and 36 healthy controls were recruited for this study. Baseline samples were collected from all subjects. For subjects with dental caries plaque was collected separately from the surfaces of three progressive stages of caries. Longitudinal samples were collected as described below. There were no significant differences between the caries and control groups for gender, race or ethnicity based on Chi-squared analysis. The distribution of race in the study population was 40% white, 38% black, and 22% other. The study population was 83% not Hispanic and 17% Hispanic, and 54% female. Ages ranged from 12 to 36 months old at enrollment (mean age was 23.6 months) and were not significantly different between the caries and control groups. There was no difference between the groups based on smoke exposure or history of antibiotic use in the previous 30 days based on Chi-squared analysis. Sample sizes were too small to analyze the effect of fluoride, since the majority of subjects (89% of caries subjects and 92% of control subjects) reported exposure to fluoride through drinking water.

### Cloning and Sequencing

An average of 54 clones (minimum 44, maximum 102) were identified per sample. The total number of clones for all samples was 9,396. A total of 6,688 clones were sequenced from baseline samples (caries and control subjects) and 2,708 clones were sequenced from longitudinal samples (caries subjects only). The shortest sequence length was 563 base pairs, and the average sequence length was 1060 base pairs.

### Taxonomic Identification and Disease Association

**Figure 1:**
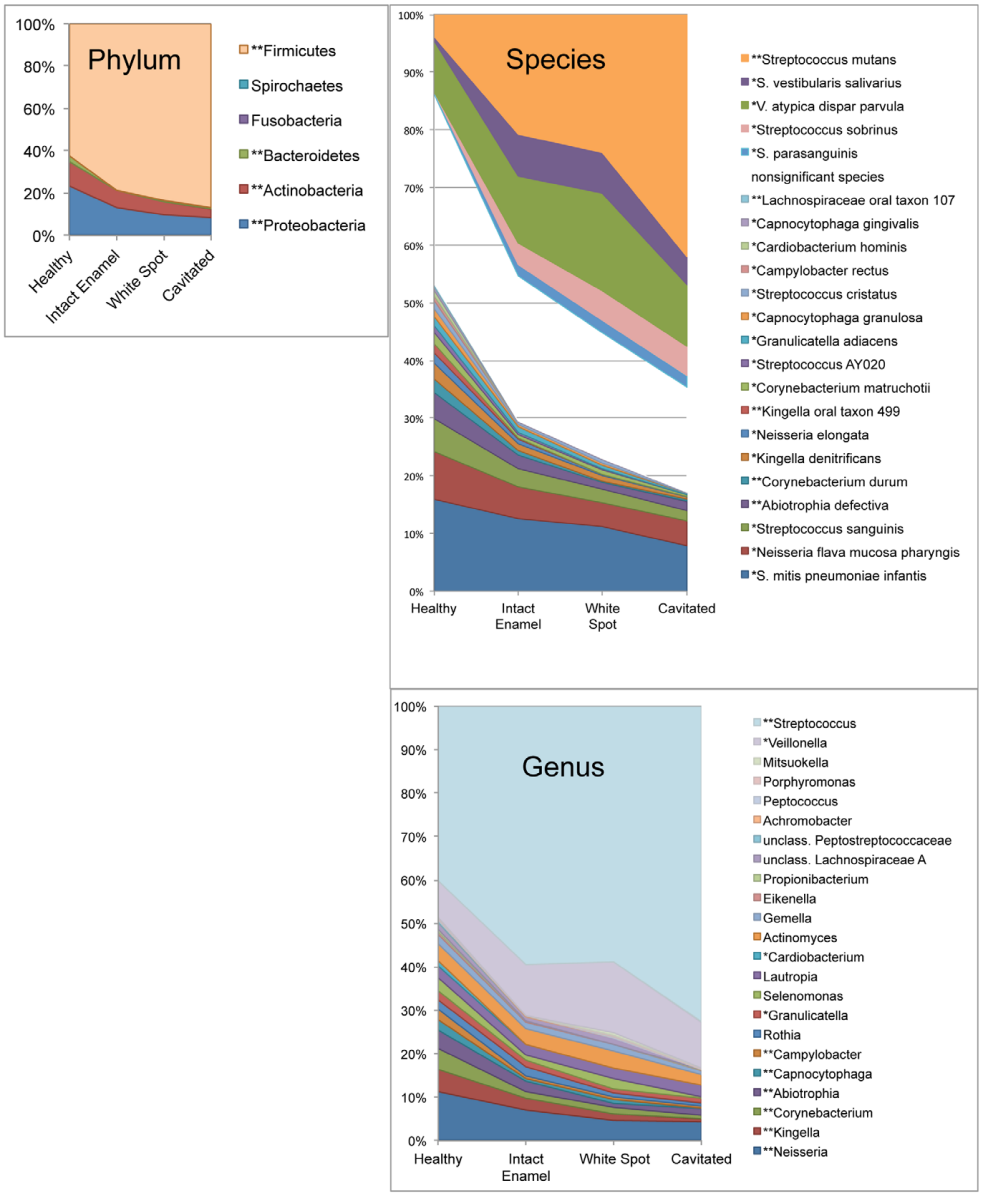
Relative levels of bacterial taxa by advancing stage of caries. Graphs at the level of phylum, genus and species are shown. Taxa are sorted by magnitude of change with stage of caries (linear mixed effects model estimates), so that taxa associated with health sort at the bottom and taxa associated with caries are shown at the top. “*” indicates taxa with *p*<0.05 and “**” indicates taxa with *p*<0.01 after the false discovery rate correction was applied. Only genera found at greater than 0.1% of total clones and species found at greater than 0.2% of total clones are shown, and only those taxa significantly associated with caries or health are shown in the species-level graph.

Overall 134 species were identified in this study, including two novel taxa. They could be assigned to 45 genera and six phyla. Only 9.65% of total clones represented uncultivated species. Baseline mean relative levels of bacterial taxa by advancing caries stage are plotted in Figure 1. At the level of phylum, *Proteobacteria*, *Actinobacteria* and *Bacteroidetes* decreased as caries stage increased, and *Firmicutes* increased. At the level of genus *Veillonella* and *Streptococcus* significantly increased with caries progression, and 8 other genera decreased as caries stage advanced (details in Figure 1). Five species-level taxa were significantly higher as caries stage increased, including *Streptococcus mutans,* the *Streptococcus vestibularis/Streptococcus salivarius* group, the *Veillonella atypica/Veillonella dispar/Veillonella parvula* group, *Streptococcus sobrinus,* and *Streptococcus parasanguinis*. Seventeen species significantly decreased as caries stage progressed (details in Figure 1).

**Figure 2:**
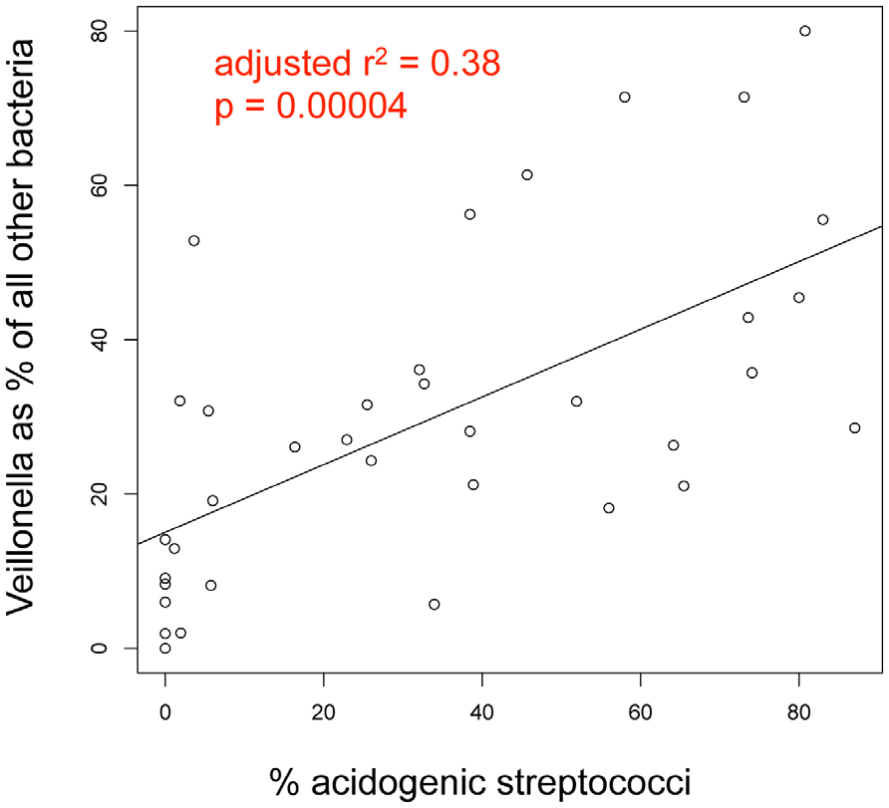
Correlation between relative levels of Veillonella and acidogenic streptococci in white spot lesions. The total % abundance of *S. mutans*, *S. sobrinus*, and *S. vestibularis/salivarius* combined is plotted against the abundance of the *Veillonella atypica/dispar/parvula* group expressed as a fraction of the remaining community. The result of a linear regression is shown as a line with the indicated parameters.

Figure 2 shows a significant correlation between the relative levels of *Veillonella* and the major acid-producing species.

### Cluster Analysis and Heterogeneity Among Subjects

**Figure 3:**
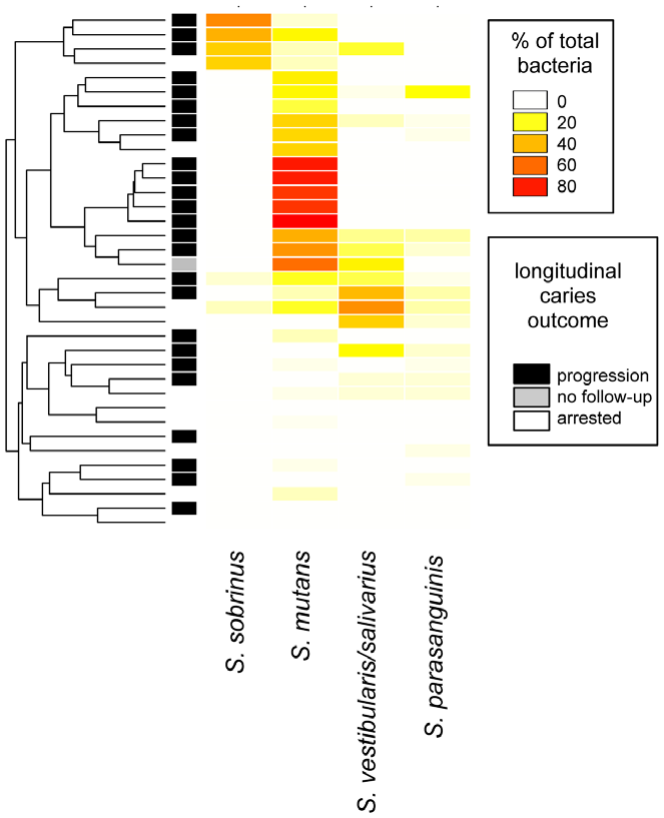
Heatmap and cluster analysis of baseline samples from white spot lesions. Abundances of those bacterial species significantly associated with caries are shown, except for Veillonella which was ubiquitous and therefore omitted. The samples (one from each of 36 subjects) are arranged by hierarchical clustering using the average method and Bray-Curtis dissimilarity. Abundance as percentage of the total community is indicated by the color scale. The bar along the left side indicates longitudinal caries activity.

Figure 3 shows an abundance heatmap with sample clustering for the significant acid producing species in baseline samples from white spot lesions. This illustrates heterogeneity among subjects with respect to the different acid producers.

### Community Diversity and Caries Stage

**Figure 4:**
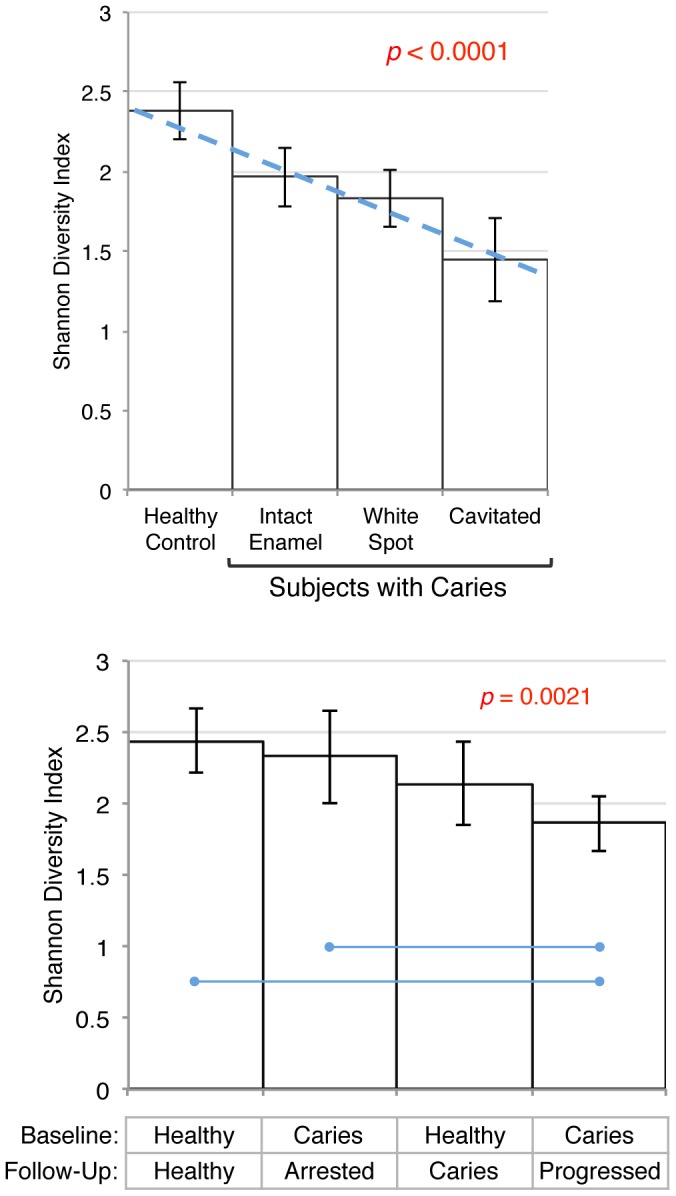
Decreasing species diversity was observed with increasing caries severity both within and among subjects. Mean Shannon Diversity Indices with 95% confidence intervals are shown. The upper panel shows diversity *within* subjects for stage of caries at baseline. Diversity was modeled using a linear mixed effects model (SAS PROC MIXED), and is shown as a dashed line (estimate =  −0.26). *Post hoc* comparisons between sample types were significant, except between white spot and cavitated lesions. The lower panel shows species diversity comparisons *among* subjects by their baseline and longitudinal caries status for samples collected from noncarious enamel (the only type of sample available from all groups) using ANOVA. Significant *post hoc* comparisons are indicated by blue lines.

Overall more taxa decreased than increased as caries stage advanced within subjects, and this was true at all phylogenetic levels. The relationship between caries stage and bacterial community diversity for baseline samples is shown in Figure 4, upper panel. Overall, bacterial diversity decreased significantly (*p*<0.0001) as caries progressed from health to cavitated lesions.

Bacterial community diversity was also significantly different among groups of subjects by ANOVA (*p* = 0.0021) as shown in Figure 4, lower panel. *Post-hoc* comparisons indicated that the samples taken from intact enamel at baseline from subjects with caries that progressed had significantly lower species diversity than that of subjects with caries that arrested and control subjects that remained healthy at follow-up.

### Bacterial Community Shifts and Caries Stage within Subjects

**Figure 5:**
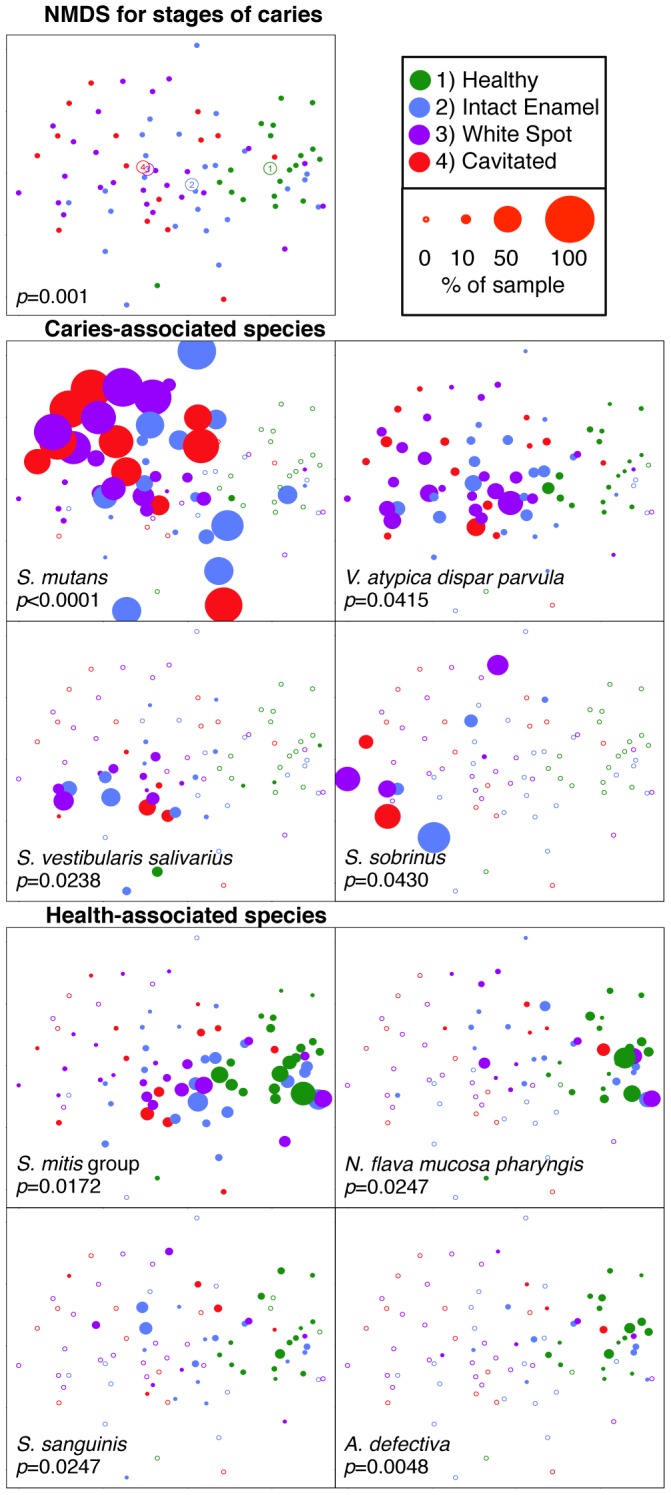
Within-subject differences in plots of a non-metric multidimensional scaling (NMDS) ordination based on Bray-Curtis Dissimilarity. Baseline samples from only the subset of subjects whose caries status remained constant over time (caries subjects that continued to develop caries and healthy subjects that remained healthy) was included, and within-subject differences by stage of caries were observed. A single sample from each stage of caries is included for each subject, and each point represents a single sample. The top panel shows the NMDS plot, with the centroid for each stage of caries marked. The metaMDS algorithm used puts the largest dimension of change along the horizontal axis. The *p*-value is for the overall ANOSIM model. The points in lower panels are sized by abundance for the most common species significantly associated with caries and health, and *p*-values are for the linear mixed effects model estimates. Empty plot symbols represent samples where that species was not detected.

Figure 5 shows a non-metric multidimensional scaling (NMDS) ordination based on Bray-Curtis dissimilarity for baseline samples from all stages of caries. A leftward shift with advancing caries stage can be observed in the plot. The ANOSIM test for all within-subject stages was significant (R = 0.232, *p = *0.001) and pairwise comparisons revealed significant differences between healthy subjects and subjects with caries for all samples: intact enamel (R = 0.266, *p = *0.002), white spot samples (R = 0.398, *p = *0.002), and cavitated samples (R = 0.538, *p = *0.002). The lower panels of Figure 4 illustrate the distribution of abundance within the samples for selected species.

### Longitudinal Analysis

**Figure 6:**
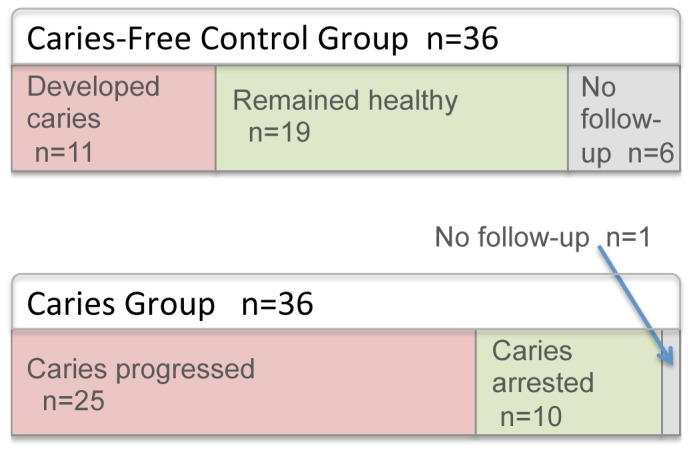
Sample sizes at baseline and outcomes at longitudinal follow-up.

Subjects were followed longitudinally to assess their ongoing caries status. Twenty-one subjects with caries returned for a follow-up visit and sampling, and chart reviews were conducted for all remaining subjects. Sample sizes according to longitudinal caries status are shown in Figure 6. The relationship of baseline microbial community composition to longitudinal clinical outcomes was examined.

**Figure 7:**
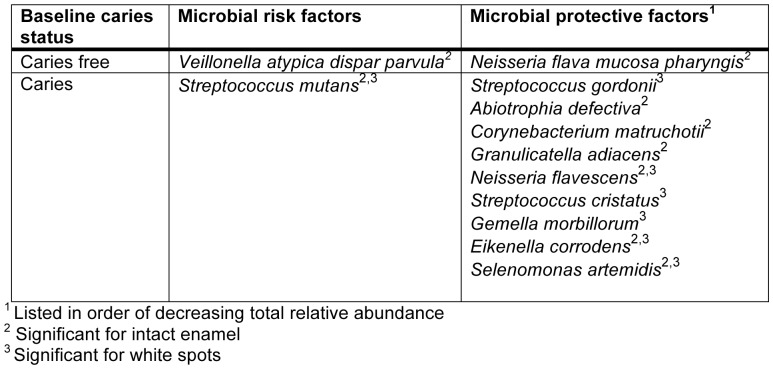
Candidate microbial risk and protective factors. Candidate microbial risk and protective factors are listed for the onset of caries in subjects that were caries free at baseline and the progression of caries in subjects that had caries at baseline.

Levels of species occurring at greater than 0.2% of the total community at baseline were compared between progressed and arrested groups for intact enamel and white spot lesions by *t-*tests. Species that significantly predicted caries and health (candidate microbial risk and protective factors) are listed in Figure 7. Only *Veillonella atypica/Veillonella dispar/Veillonella parvula* remained significant after a false discovery rate correction was applied.

**Figure 8:**
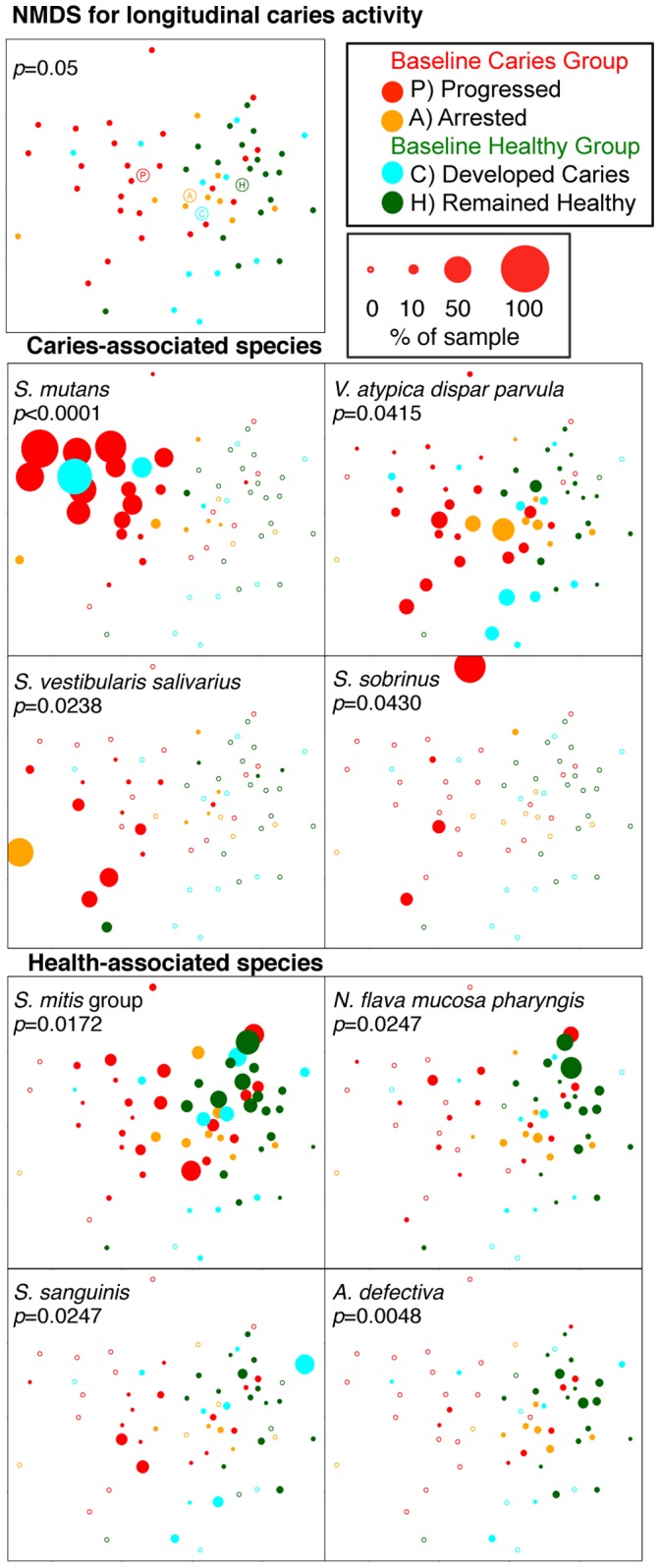
Among-subject differences visualized by non-metric multidimensional scaling (NMDS) ordination based on Bray-Curtis Dissimilarity. Among-subject differences by longitudinal caries activity were observed. The plots represent baseline community profiles for healthy subjects and subjects with caries. The baseline patient class and subsequent disease activity are color-coded. Samples from healthy enamel were the only stage available from all subjects and so were used here. The top panel shows the NMDS plot, with the centroid for each clinical group marked. The *p*-value is for the overall ANOSIM model. The points in lower panels are sized by abundance for the most common species significantly associated with caries and health, and *p*-values are for the linear mixed effects model estimates.

The NMDS plots shown in Figure 8 are keyed by longitudinal clinical status. ANOSIM analysis was significant for the overall model (R = 0.06819, *p = *0.05), and pairwise comparisons revealed a significant difference between control subjects that remained healthy and those that developed caries (R = 0.1908, adjusted *p = *0.015), as well as a difference between control subjects that remained healthy and caries subjects that progressed (R = 0.1571, adjusted *p = *0.015).

Repeat samples were obtained for 21 subjects with caries, and exploratory analyses were conducted to examine changes over time for major species. No differences in relative levels of species were observed over time for any stage of caries or for caries progressions for any of the species associated with advancing stage of caries within subjects (*S. mutans, S. vestibularis/S. salivarius, V. atypica/V. dispar/V. parvula, S. mitis/S. pneumoniae/S. infantis/S. oralis, N. flava/N. mucosa/N. pharyngis, S. sobrinus, S. sanguinis,* or *A. defectiva*). The comparison of Bray-Curtis dissimilarity over time within individuals revealed significantly greater similarity between samples from the same individual than between samples from different individuals, an indicator of long-term stability.

## Discussion

For this study of the initiation of caries in young children, cross-sectional samples from dentally healthy children and from children with very early-stage caries were collected. Samples were collected from intact, noncarious tooth enamel from all subjects. Samples were also collected from two defined stages of caries from the subjects with the disease: from white spot lesions (the initial presentation of caries) and from cavitated lesions (more advanced and irreversible stage of caries). Subjects were followed longitudinally to determine progression of caries, and longitudinal samples were collected from those subjects who returned for follow-up. All subjects were given preventive counseling and treated with fluoride, yet a high proportion of subjects in both the caries group and the healthy group subsequently developed new lesions (Figure 6).

Bacterial community analysis was conducted using 16S rRNA gene cloning and sequencing, and bacteria were identified using an oral 16S rDNA database specifically curated for clinical data [17]. Bacterial taxa were measured as a percent of the total community. This open-ended approach allowed a comprehensive look at the bacterial communities associated with caries and dental health in children.

### Caries-associated Species

#### Heterogeneity among samples

Figure 3 shows a cluster analysis for subjects with caries, and illustrates distinctive and heterogeneous profiles. As expected, the majority of subjects with caries exhibited high levels of *S. mutans*. However, perhaps the most interesting finding is that, as previously observed [11]–[13], [18], [19], not all subjects harbored high levels of *S. mutans*. In the present study two additional profiles, one dominated by *S. sobrinus* and one by *S. vestibularis/S. salivarius*, were observed. When present, one of these three species appeared to dominate the community, suggesting functional redundancy and competition rather than cooperative consortia. This has considerable clinical significance for diagnostic and therapeutic purposes. Interventions for caries have been focused almost entirely on *S. mutans*. It appears this target is appropriate for many, but not all, children. In addition, it seems possible that any of these three species could provide functional redundancy (lactate production and caries) if the others were eliminated by vaccination or other targeted therapy. In fact, one caries-active subject in the present study appeared to undergo a shift from an *S. mutans*-dominated profile to *S. sobrinus*, and another from an *S. salivarius/S. vestibularis*-dominated profile to *S. mutans* over time (data not shown). This potentially important observation will need to be investigated further in future studies. In other studies, additional species have been associated with disease when *S. mutans* was absent, including *Lactobacillus* species [11], [12], *Bifidobacterium dentium* [11], and *Scardovia wiggsiae* [15]. None were detected in significant levels in the current study, and this could be attributed to differences in study populations, severity of caries at the time of sampling, and methodologies, notably culture versus DNA-based methods.

For several of the samples taken from white spot lesions, a dominant, acid-producing species was not identified (Figure 3). About half of these subjects did not show progression of the white spot lesions observed at baseline (shown in dark bars in left column, Figure 3), suggesting that their lesions may have been developmental hypomineralizations rather than acid-induced demineralizations. These are difficult to distinguish clinically. In the remaining subjects, *S. mutans*, *S. sobrinus* or *S. vestibularis/S. salivarius* were all detected at low levels in other samples from the same subject. It was difficult to precisely sample these very young and often uncooperative subjects, and the target lesion may have been missed. It is also possible that genetically distinct strains of bacteria grouped with innocuous species by 16S rRNA analysis were the culprits, since the oral streptococci show high variability of their total genomes relative to their 16S rRNA genes [20].

##### S. mutans

*S. mutans* is highly acidogenic and aciduric, and considerable clinical and laboratory data implicates this species as the primary pathogen in human dental caries (reviewed in [10], [21], [22]). This role was corroborated in the present study. *S. mutans* was the most abundant species observed in samples from children with caries, and levels were highly significantly associated with lesion stage (Figure 1). It was also identified as a candidate risk factor for caries progression. Mean levels of *S. mutans* were higher in both intact enamel and white spot samples for subjects who developed new lesions as compared to subjects who did not (Figure 7). Although levels of *S. mutans* did not significantly predict the development of caries in previously healthy subjects, just two healthy control samples showed high levels of *S. mutans* at baseline (see *S. mutans* panel on Figure 8), and both of these subjects subsequently developed caries.

##### S. sobrinus

*S. sobrinus* is closely related to *S. mutans,* and these species are often referred to collectively as the mutans streptococci [20]. *S. sobrinus* was associated with caries in the current study (Figure 1), and appeared to be the primary pathogen in some subjects (Figure 3). Caries remained active over time in most of these subjects as shown in Figure 3, and *S. sobrinus* levels remained high in longitudinal samples (data not shown). In one subject with active caries *S. mutans* was replaced by *S. sobrinus* over time. In most samples dominated by *S. sobrinus*, low levels of *S. mutans* were present as well. Unlike *S. mutans*, it was not detected in any healthy control subjects, suggesting it may be a more specific predictor of disease than *S. mutans*.

The pathogenic potential of *S. sobrinus* has been established in a rat model [23], and it has been associated with childhood caries in several investigations from different geographic locales [13], [24]–[30]. It has often been found as a co-colonizer with *S. mutans*, and co-colonization has been consistently associated with greater caries risk and stronger association with caries [13], [24]–[30].

#### Salivarius group

*S. vestibularis* and *S. salivarius* are members of the salivarius group [31] and have been distinguished from each other based on phenotype. However, they cannot be reliably differentiated based on 16S rDNA sequence [17], and therefore the taxon designation here includes both species. Both species have been isolated from the human oral cavity, and in animal studies, isolates of *S. salivarius* have been shown to be strongly cariogenic, although less so than *S. mutans* [32]–[35]. In vitro studies suggest *S. vestibularis* is only mildly cariogenic [35], [36]. *S. salivarius* has been associated with caries in clinical studies using DNA-based methods [11], [37].

In the present study the salivarius group was significantly associated with caries, and appeared to be the primary pathogen in some subjects (Figure 3). The sample size is too small for analysis, but it appeared caries was less likely to remain active in these subjects than for those with an *S. mutans* or *S. sobrinus*-dominated profile as shown in Figure 3. However, *S. salivarius* levels remained high in most subjects with this profile who did progress (data not shown), providing support for a role in caries.

On the other hand, some strains of *S. salivarius* are known to produce urease, which hydrolyzes urea to ammonia, and so may be caries-protective [23], [38], [39]. A recent *in vivo* study found increased *S. salivarius* urease activity in plaque following a sucrose challenge [40], and *S. salivarius* has been associated with health in one study [41]. These conflicting findings might be explained by differences in detection methodology or genomic differences that are not detected using 16S rRNA methodologies. Future studies using higher resolution molecular techniques are needed to study this heterogeneous group of bacteria.

##### Streptococcus parasanguinis

In the present study *S. parasanguinis* was significantly associated with caries (Figure 1), but was not a dominant member of the community for any of the subjects, even those in whom no *S. mutans* was detected (Figure 3). *S. parasanguinis* has been significantly associated with caries in young children in two previous studies that utilized 16S rRNA methods [15], [37], and in another similar study it was found at high levels in an *S. mutans*-free subject with caries [11]. It ferments multiple carbohydrates to lactate and other organic acids [42], [43], and it appears to be moderately acid tolerant [44], consistent with a role in dental caries. However, it has also been associated with health in one study [41], and it has been reported to hydrolyze arginine to ammonia, and not to produce extracellular polysaccharide from sucrose [42]. So the contribution of *S. parasanguinis* to dental caries deserves further study.

#### Lactobacilli

Lactobacilli were rarely detected, and when present were at very low levels in this cohort of young children with the earliest stages of caries. Lactobacilli have shown a robust association with more advanced caries in many studies [11], [45]–[47] and in older children using a very similar experimental approach to the one used here [12], strongly suggesting that they are a later colonizer in microbial succession as caries-associated communities mature and shift to a lower pH.

##### Veillonella atypica/Veillonella dispar/Veillonella parvula

Three species of *Veillonella* have regularly been found in the oral cavity. These species have been distinguished from each other based on phenotype, but they cannot be reliably differentiated based on 16S rDNA sequence [17], [48], and therefore the taxon designation here includes all three *Veillonella* species. *Veillonella* rely solely on lactate and other organic acids as an energy source [49]. *Veillonella* was detected in most samples in the current study, and it was higher in samples from caries lesions (Figure 1, *p = *0.0415, significant by linear mixed effect modeling). *Veillonella* was found to be significantly associated with caries in children in previous molecular studies as well [11], [15], [29], [37], [50]. *Veillonella* was highly correlated with the total of all known acid producing species, as shown in Figure 2. This is not surprising given its reliance on lactate as its nutrient source, and has potential clinical utility since *Veillonella* levels may serve as a sensitive biologic indicator and early warning of acid production. Among children without previous history of caries, *Veillonella*, but not *S. mutans* or other acid-producing species, predicted future caries (Figure 7). These findings need to be corroborated in a larger clinical study, and could lead to useful risk assessment methods for caries.

The contribution of *Veillonella* to caries in in vivo studies has been somewhat unclear, with laboratory studies showing effects on pH in both directions [51], [52]. Chemostat studies have shown levels of *Veillonella* species to increase as pH falls following glucose adminstration [53]–[56]. Mounting clinical data associating elevated levels of *Veillonella* with caries [11], [12], [15], [50], however, suggests that increasing levels of *Veillonella* do not halt caries by raising pH. Taken together with in vitro data [57] it appears *Veillonella* may even facilitate further acid production by *S. mutans* or other species by removing lactate from the environment and creating a higher pH microenvironment. A recent in vivo study showed that *Veillonella* mitigated the inhibitory effects of *S. gordonii* on *S. mutans* sugar metabolism [58], suggesting a specific interaction between *S. mutans* and *Veillonella* that may be more complex than pH.

### Health-associated Species

A large number of species were associated with health in the current study. These species may be beneficial, and potential mechanisms are discussed below. Seventeen species were found at significantly higher relative levels in health (Figure 1) as compared to the more advanced stages of caries, with the major contributors being the *Streptococcus mitis/Streptococcus pneumoniae/Streptococcus infantis/Streptococcus oralis* group, the *Neisseria flava/Neisseria mucosa/Neisseria pharyngis* group, and *Streptococcus sanguinis*. At the level of phylum the *Proteobacteria*, *Actinobaceria* and *Bacteroidetes* were all health associated (Figure 1).

Several species were identified as candidate microbial protective factors against caries onset or progression from the longitudinal data as well (Figures 7 and 8). Baseline levels of the *Neisseria flava/Neisseria mucosa/Neisseria pharyngis* group were higher in control subjects that remained healthy when compared to control subjects that subsequently developed caries, suggesting a beneficial role in the caries process. *Streptococcus gordonii, Abiotrophia defectiva, Corynebacterium matruchotii, Granulicatella adiacens, Neisseria flavescens, Streptococcus cristatus, Gemella morbillorum, Eikenella corrodens,* and *Selenomonas artemidis* were all found at higher levels in subjects with caries that did not progress as compared to those whose caries progressed, suggesting a beneficial role for these species as well. Many of these candidates have been associated with health in previous clinical studies. Becker et al. found high levels of *A. defectiva* in a healthy subject and high levels of *C. matruchotii* in the intact enamel sample of a subject with caries. *A. defectiva* [29], [41], *S. cristatus* [11], [18], [29], [41], *S. gordonii* [59], and *G. morbillorum* [11], [41] were associated with health in previous investigations. *N. flavescens* was associated with health using next-generation sequencing and saliva samples [60]. *N. flavescens* is asaccharolytic [61]. *S. cristatus* and *S. gordonii* catabolize arginine to ammonia [62], [63] potentially raising pH. *S. gordonii* also inhibits biofilm formation [64] and bacteriocin production [64], [65] by *S. mutans. C. matruchotii* can utilize lactate [66], so it may also raise pH. *S. artemidis* produces propionic and acetic acids [67], which may be less destructive than lactic acid [68], [69]. Further laboratory study is required to determine the ability of these candidate species to protect against caries onset and progression.

### Decreasing Species Diversity in Caries

In the present study diversity decreased with increasing caries stage within subjects, and among subjects was lowest in the group of subjects with caries that progressed (Figure 4). This confirms previous investigations that associated caries with a reduction in species diversity [12], [70]–[72] (Figure 4), and supports what has been called the “ecological plaque hypothesis” [57]. This explains caries as the result of an ecologic disruption of the normal, healthy bacterial community that occurs when carbohydrates are frequently available and acid-producing species lower pH through glycolytic activity. As a result acid-sensitive species are eliminated, and communities become dominated by just a few highly acid-tolerant species. This may accelerate further as species that normalize pH by the production of ammonia are lost [16], and this loss of diversity may have implications for bacterial community resilience and restoration of oral health.

### Sequence Identification

Technical limitations of the current study include the small number of clones that were identified from each sample, and the “universal” primers used for 16S rRNA gene amplification that, although used with low stringency, may underrepresent some taxa. Degenerate universal primers have recently been developed and tested for multiple regions of the 16S rRNA gene that can be used in future studies to improve representation of community composition. Because oral streptococci show relatively low diversity in 16S rDNA sequence, longer reads are required to achieve resolution at the species level. Mean read length was 1060 bp, considerably longer than current next generation sequencing methodologies achieve. Future studies targeting the most highly variable regions of the 16S rRNA gene with optimized read lengths are needed to bring the power of deep sequencing approaches to understanding caries-associated bacterial communities.

## Summary and Conclusions

Bacterial community profiles associated with the onset of early childhood caries in the young primary dentition were compared to bacterial communities found on healthy teeth and in dentally healthy children using a combination of a cross-sectional design representing the various stages of caries, and longitudinal clinical sampling. Differences between health and disease were observed at all taxonomic levels including phylum, genus and species. As expected, *S. mutans* was the dominant species in many, but not all, subjects with caries. Elevated levels of *S. vestibularis/S. salivarius, S. sobrinus*, and *S. parasanguinis* were significantly associated with caries, and were observed at especially high levels in subjects with little or no *S. mutans*, suggesting these species are alternative pathogens. *Veillonella*, which metabolizes lactate, was associated with caries and was highly correlated with total acid producing species. Among children without previous history of caries, *Veillonella*, but not *S. mutans* or other acid-producing species, predicted future caries. Bacterial community diversity was reduced in caries as compared to health, as many species appeared to occur at lower levels or be lost as caries advanced, including the *Streptococcus mitis/Streptococcus pneumoniae/Streptococcus infantis/Streptococcus oralis* group, the *Neisseria flava/Neisseria mucosa/Neisseria pharyngis* group, and *Streptococcus sanguinis*. This may have implications for bacterial community resilience and the restoration of oral health.

## Materials and Methods

### Ethics Statement

Approval from the Nationwide Children’s Hospital Institutional Review Board was obtained for this protocol, and written consent was obtained from the parents of all subjects.

### Clinical Methods

#### Subject recruitment

Subjects with dental caries and a dentally healthy control group were recruited from the Nationwide Children’s Hospital Dental Clinic in Columbus, Ohio. General exclusionary criteria for either group included (i) age greater than 36 months, (ii) indications for infective endocarditis prophylaxis, and (iii) professional cleaning in the past 30 days. Only one child per family was included in each group. The inclusion requirement for the caries group was the presence of at least two maxillary incisors with white spot lesions, no cavitated lesion greater than 1 mm, and no existing restorations. Subjects with caries were asked to return every four to six weeks. Age-, race-, and gender-matched healthy control subjects that were caries-free and had no existing restorations were also recruited.

#### Sampling and clinical data collection

For the healthy subjects dental plaque was sampled from healthy enamel (stage 1). For the subjects with dental caries plaque was collected separately from the surfaces of each of three progressive stages: 2) intact enamel, 3) white spot lesions, and 4) cavitated lesions, if present. Therefore one sample was collected from each healthy subject, and either two or three samples were collected from each subject with caries, depending on whether cavitated lesions were present. For subjects with caries, all carious surfaces were scored. Dental plaque was collected by swiping the tooth or lesion surface with a coarse endodontic paper point. Each plaque sample was obtained by pooling from multiple teeth. Samples were placed in a sterile 1.5-ml microcentrifuge tube and frozen for storage.

A subset of caries subjects returned for a follow-up visit, during which the progression of any white spot or cavitated lesion was scored and the subject was re-sampled as above. Following the conclusion of the clinical study, longitudinal caries status for all healthy subjects and caries subjects who did not return for follow-up was determined by chart review.

During the first visit, a brief written survey regarding antibiotic history, fluoride status, and exposure to cigarette smoke was completed by the parent. At every visit, subjects received a toothbrush prophylaxis and fluoride varnish was applied. Parents received oral hygiene instructions and anticipatory guidance regarding the contribution of dietary factors to caries onset and progression.

### Laboratory Methods

#### Sample preparation

Bacterial DNA was isolated using a bead beater as previously described [12].The bacterial DNA was purified using glass beads as previously described [73] and frozen until analysis.

#### PCR amplification, cloning, and sequencing

The 16S rRNA genes were amplified from the purified bacterial DNA, and the PCR products were cloned and sequenced as previously described [74]. Briefly, universal primers 5′-GTT TGA TCC TGG CTC AG-3′ (forward) and 5′-AAG GAG GTG ATC CAG CC-3′ (reverse) were used and the PCR products were examined by electrophoresis in 1% agarose and purified using the QIAquick PCR Purification Kit (QIAGEN, Valencia, CA). Amplicons were cloned into *E. coli* using a TOPO TA cloning kit (Invitrogen, San Diego, CA). Amplicons included the 16S rRNA gene hypervariable regions V5–V9.

### Bacterial 16S rDNA Sequence Identification

Sequences from each clone were identified by comparing it to a local, curated oral microbiome database [17], found at http://microbiome.osu.edu, using BLAST [75]. Clinical sequences were required to be ≥98.0% similar to species-level taxonomic units in the database for identification. Sequences with similarity scores <98% were further analyzed by BLAST against the GenBank *nr/nt* database, the results manually examined, and chimeric sequences removed. Sequences that were observed only once and failed to match sequences in GenBank were not considered. Remaining novel sequences were added to the database.

### Data Management and Statistical Analyses

Chi-squared analysis was used to compare the caries and control groups by gender, race, and ethnicity. A *t-*test was used to compare the two groups by age.

Levels of each species were calculated as a percent of total bacteria for each sample. Mean relative levels and 95% confidence intervals were determined for the most prevalent species using JMP (JMP, Version 7.0, SAS Institute Inc., Cary, NC). Repeated measures analysis was performed using PROC MIXED in SAS (SAS Institute Inc., SAS 9.2, Cary, NC) using the default structure. The sequential stages of health/caries from which samples were collected were assigned a numeric value and used in the PROC MIXED analysis. Using this scale, healthy control samples were assigned a value of 1, intact enamel samples from subjects with caries 2, white spot lesions 3, and cavitated lesions 4. A linear mixed effects model in PROC MIXED was used to calculate an estimate of the percent change in relative level for each taxon for caries stages 1 through 4, with α = 0.05, and the false discovery correction was applied.

Species abundance matrices were generated based on the BLAST classification of sequences. These matrices were then used to calculate the Bray-Curtis dissimilarity between each pair of samples. This statistic has been widely used in ecology to measure differences between communities and has a number of desirable properties, including giving sensible results for abundance matrices with many zeros and often showing good correlations with underlying environmental factors [76]. The calculation was performed with the *vegdist* function of the *vegan* package of the R programming language [76]. Direct comparisons were done on the Bray-Curtis matrix using the function *t.test* of the *stats* package of R [77], and ordinations were performed by non-metric multidimensional scaling (NMDS) using function *metaMDS* of the *vegan* package [76]. The centroids of points for the four different stages of caries/health were calculated. ANOSIM, a permutation-based test to measure differences between sample groups, was performed using the PRIMER 6 program (PRIMER-E, Plymouth, UK). Shannon diversity was calculated using a short R script that applied functions *rrarefy* and *diversity* from the *vegan* package [76], and was analyzed by SAS PROC MIXED for comparisons by severity and ANOVA for comparisons by subject group. Samples contained a varying number of sequences, with 44 being the smallest number for a sample that was included in the analysis. A random selection of 44 sequences was taken and the Shannon Diversity Index was calculated 10 times for each sample, and the average of these 10 indices was used for further analysis.

Longitudinal data was analyzed by calculating the Bray-Curtis dissimilarity matrix for subjects that were sampled at two time points, and the mean dissimilarities between samples from the same patient and samples from different subjects were compared by *t-*tests not assuming equal variance using the function t.test in stats package of R [77]. Longitudinal data was also explored at the species level. Mean abundance of the most abundant and significant species was compared for baseline and longitudinal samples using paired *t-*tests. Comparisons were made at each level of severity, and were tested for all caries samples, progressed samples only, and arrested samples only. *t-*tests were also used to compare progressed and arrested samples by the percent change in mean levels of these species between sampling times for enamel and white spot lesions.

*t-*tests and paired *t-*tests for *post hoc* comparisons were computed in JMP. The False Discovery Rate correction for multiple comparisons was determined using SAS.

#### Sample clustering and heatmap analysis

We generated heatmaps to graphically display species abundances for the most informative caries-associated species. Bray-Curtis dissimilarities between samples were determined using the species/sample abundance matrix for all white spot samples using function *vegdist* of vegan package in R [76], and hierarchical clustering of the samples was carried out with an average method using the function *hclust* of stats package in R [77]. The resulting dendrogram was reordered by assigning weights of 0 to the arrested samples and 1 to the progressed and using the mean as the agglomerative function. The reordered dendrogram and the abundance matrix for 4 caries-related species was given as input to the heatmap command of R.

#### Linear regression analysis of *Veillonella atypica/Veillonella dispar/Veillonella parvula* abundance

The combined abundance of the acidogenic species *S. mutans*, *S. sobrinus*, and *S. vestibularis/S. salivarius* was calculated for each white spot lesion sample. The abundance of *V. atypica/V. dispar/V. parvula* was calculated as fraction of the remaining organisms and the relationship between the two values was modeled by linear regression using command *lm* of R [77].
